# Respiratory Membrane *endo*-Hydrogenase Activity in the Microaerophile *Azorhizobium caulinodans* Is Bidirectional

**DOI:** 10.1371/journal.pone.0036744

**Published:** 2012-05-15

**Authors:** Brittany N. Sprecher, Margo E. Gittings, Robert A. Ludwig

**Affiliations:** Sinsheimer Laboratories, Department of Molecular, Cellular and Developmental Biology, University of California Santa Cruz, Santa Cruz, California, United States of America; Royal Netherlands Institute of Sea Research (NIOZ), The Netherlands

## Abstract

**Background:**

The microaerophilic bacterium *Azorhizobium caulinodans,* when fixing N_2_ both in pure cultures held at 20 µ*M* dissolved O_2_ tension and as endosymbiont of *Sesbania rostrata* legume nodules, employs a novel, respiratory-membrane *endo-*hydrogenase to oxidize and recycle endogenous H_2_ produced by soluble Mo-dinitrogenase activity at the expense of O_2_.

**Methods and Findings:**

From a bioinformatic analysis, this *endo*-hydrogenase is a core (6 subunit) version of (14 subunit) NADH:ubiquinone oxidoreductase (respiratory complex I). In pure *A. caulinodans* liquid cultures, when O_2_ levels are lowered to <1 µ*M* dissolved O_2_ tension (true microaerobic physiology), *in vivo endo*-hydrogenase activity reverses and continuously evolves H_2_ at high rates. In essence, H^+^ ions then supplement scarce O_2_ as respiratory-membrane electron acceptor. Paradoxically, from thermodynamic considerations, such hydrogenic respiratory-membrane electron transfer need largely uncouple oxidative phosphorylation, required for growth of non-phototrophic aerobic bacteria, *A. caulinodans* included.

**Conclusions:**

*A. caulinodans in vivo endo*-hydrogenase catalytic activity is bidirectional. To our knowledge, this study is the first demonstration of hydrogenic respiratory-membrane electron transfer among aerobic (non-fermentative) bacteria. When compared with O_2_ tolerant hydrogenases in other organisms, *A. caulinodans in vivo endo*-hydrogenase mediated H_2_ production rates (50,000 pmol 10^9^·cells^−1^ min^−1^) are at least one-thousandfold higher. Conceivably, *A. caulinodans* respiratory-membrane hydrogenesis might initiate H_2_ crossfeeding among spatially organized bacterial populations whose individual cells adopt distinct metabolic states in response to variant O_2_ availability. Such organized, physiologically heterogeneous cell populations might benefit from augmented energy transduction and growth rates of the populations, considered as a whole.

## Introduction

Given the relatively low (−414 m*V*) potential in aqueous solution for the standard hydrogen bio-electrochemical half-cell, hydrogen gas (H_2_) is a strong electron (e^–^) donor, whereas in the back-reaction, combining H^+^ ions are weak e^–^ acceptors. Hydrogenases, which catalyze this reaction, are widely distributed among bacteria [1]. Diverse aerobic bacteria employ O_2_-tolerant (group-1) hydrogenases as e^–^ donor, oxidizing substrate H_2_ at the expense of substrate O_2_ as preferred e^–^ acceptor, driving oxidative phosphorylation. These group-1 hydrogenases, both soluble and membrane-associated, typically include globular, heterodimeric catalytic proteins. In the stably membrane-associated group-1 hydrogenases, each catalytic heterodimer is first exported and then stably complexes with a membrane-integral diheme *b-*type cytochrome [2]. Resulting heterotrimeric complexes are typically denoted ‘uptake hydrogenases’ as they operate *in vivo* as unidirectional catalysts of H_2_ oxidation. Product H^+^ ions, released on the exterior (*exo*) face of cell membranes, directly contribute to trans-membrane proton-motive force absolving these activities of any, obvious chemiosmotic (ion-pumping) workload. These stably membrane-associated, group-1 heterotrimeric complexes may be termed *exo*-hydrogenases.

The reversible or H_2_ evolving (group-4) hydrogenases, also membrane-associated, are encoded by completely divergent gene-sets. The group-4 hydrogenases are typically employed by anaerobic bacteria to produce H_2_ as fermentative end-product and in so doing, facilitate overall cellular oxidation-reduction balance [1]. In anaerobes, the hydrogenesis (H_2_ production from H^+^ ions) reaction involves direct coupling of group-4 hydrogenases as e^–^ acceptor with various e^–^ donors such as formate and carbon monoxide dehydrogenases as membrane-integral complexes [3], [4]. In contrast to group-1 membrane-associated uptake hydrogenases, the catalytic heterodimers of group-4 hydrogenases are oriented to the cytosolic (*endo*) face of cell membranes [5], [6] and so may be termed *endo*-hydrogenases.

However, *endo*-hydrogenases are not exclusive to fermentative anaerobes. We recently reported on a novel *endo*-hydrogenase in the aerobic microaerophile *Azorhizobium caulinodans* which requires oxidative phosphorylation for growth. Indeed, *A. caulinodans* employs both membrane-associated *exo*- and *endo*-hydrogenases when respiring with H_2_ as e^–^ donor. In chemolithotropic cultures with exogenous H_2_ as sole energy source, *A. caulinodans* primarily relies on *exo-*hydrogenase activity [7]. Archetype *A. caulinodans* strain ORS571 was originally isolated as N_2_-fixing endosymbiont of stem- and root- nodules in *Sesbania rostrata*, an annual legume indigenous to the Atlantic coastal Sahel [8]. *A. caulinodans* ORS571 may be cultured diazotrophically (N_2_ as sole N-source) and organotrophically (oxidizable organic acids as C- and energy source) under a reduced (2%) atmosphere [9]. Its sole N_2_ fixing activity, Mo-dinitrogenase, also produces stoichiometric H_2_ in an ATP-dependent process [10], [11]. In such diazotrophic liquid cultures, respiratory-membrane uptake hydrogenase activity allows input of endogenous H_2_ as fuel for oxidative phosphorylation, recovering invested ATP. In contrast to use of exogenous H_2_, in endogenous H_2_ uptake, *endo*-hydrogenase activity predominates [7]. In *Sesbania rostrata* (legume) nodules actively fixing N_2_, *A. caulinodans* endosymbionts employ both *exo*- and *endo*-hydrogenases to recycle endogenous H_2_ produced by Mo-dinitrogenase activity [12]. In these cases, both *exo*- and *endo*-hydrogenases function as uptake hydrogenases.

However, as we demonstrate here, *A. caulinodans endo*-hydrogenase *in vivo* activity is bidirectional, reversing in response to physiological O_2_ availability. Given sufficient O_2_, *endo*-hydrogenase operates in H_2_ uptake mode. Under strict O_2_ limitation, *endo-*hydrogenase reverses and operates in hydrogenesis mode at extraordinarily high *in vivo* rates. Hitherto, *endo*-hydrogenase mediated hydrogenesis has been masked as it occurs in N_2_ fixing pure cultures in which Mo-dinitrogenase activity itself also produces H_2_ [10], [11]. Because *exo*-hydrogenase invariably operates in H_2_ uptake mode, *exo*- and *endo*-hydrogenases then function at cross-purposes, yielding a novel and seemingly paradoxical physiology.

## Results

### Hyq *endo*-hydrogenase is a Core Homolog of L-type Respiratory Complex I

**Figure 1:**
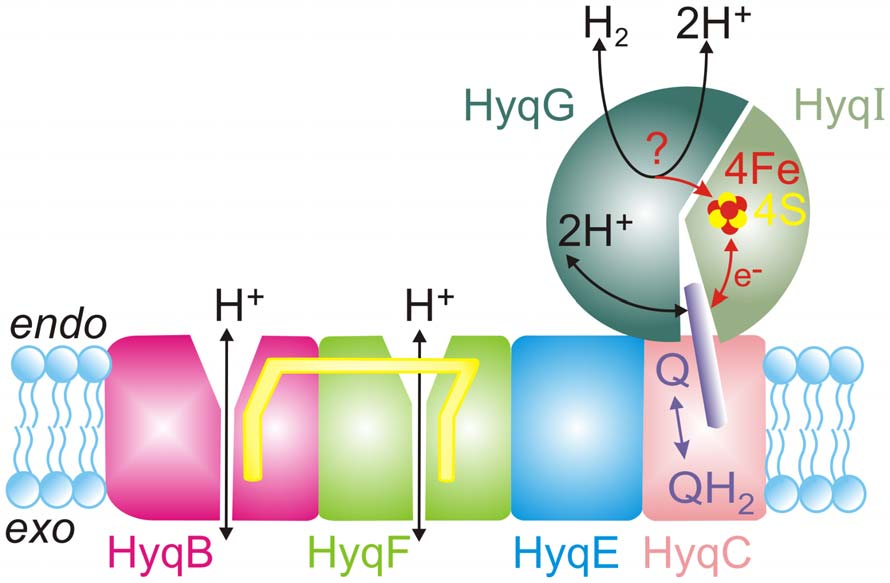
Structure-function rendering of L-type Hyq *endo*-hydrogenase by analogy and homology to respiratory complex I. Inferred membrane ubiquinone (Q) or ubiquinol (QH_2_) binding at the interface of HyqC, HyqG and Hyq I requires partial (14Å) extraction from the respiratory membrane hydrophobic phase; yellow rods represent linked transmembrane and transverse α-helices [14]. Any HyqG catalytic site remains speculative; *in vivo* activity is in principle fully reversible (see Discussion).

The *Azorhizobium caulinodans hyq^+^* operon (Entrez Gene identifier: AZC4360–AZC4355) encodes an *endo*-hydrogenase including six discrete structural proteins as well as a transcriptional activator [7]. From SUPERFAMILY analysis, a hidden Markov model library of protein structures [13], the six catalytic *A. caulinodans* Hyq proteins all have close homologs among the Nuo proteins of NADH:quinone oxidoreductase, commonly referred to as ‘L-type’ respiratory complex I (Table 1). Bacterial respiratory complex I typically includes 14 subunits equally divided into membrane-integral (L_O_) and cytosol-interfacing, membrane-peripheral (L_1_) subcomplexes [14], [15]. In pairwise primary amino acid sequence alignments, four Hyq proteins (HyqBCEF) and four L_O_ subcomplex NuoHJLM proteins are ∼60% conserved (Table 1; Figs. S1, S2, S3, S4, S5, S6). For the L_1_ subcomplexes, two (HyqGI) proteins are homologs of three (NuoCDB) proteins. From SUPERFAMILY analysis, HyqG corresponds to a fused NuoC::D (SSF56762). The HyqG (504 residues) N-terminal domain (residues 1–156) is homologous to NuoC, and its C-terminal domain (residues 157–504) is homologous to NuoD. Because its (SSF56762) superfamily also includes the group-1 hydrogenase catalytic (large) subunit, HyqG together with HyqI presumably catalyze hydrogenase activity. HyqI, a small FeS protein, is a NuoB (SSF56770) homolog; all HyqI orthologs show conserved cys-55, cys-58 (Cys-X-X-Cys), cys-112 and cys-152 residues likely coordinating a N2-type, high-potential 4Fe4S center, which in respiratory complex I serves as immediate e^–^ donor to membrane quinone [16], [17]. The binding site for complex I membrane quinone, its e^–^ acceptor, is a cavity formed between a four-helix bundle of NuoD, the H1 helix of NuoB, and transmembrane helix 1 of NuoH [14], [15], [18], all of which elements are conserved in Hyq *endo-*hydrogenases (Table 1; Figs. S1, S2, S3, S4, S5, S6). By inference, the Hyq *endo*-hydrogenase of microaerophiles constitutes a core L-type H_2_:ubiquinone oxidoreductase (Fig. 1).

**Table 1:** *A. caulinodans* Nuo (NADH:quinone oxidoreductase) and Hyq (*endo*-hydrogenase) structural homologs.

*A. caulinodans* complex I	EntrezGene identifier	*T. thermophilus* complex I	*A. caulinodans* hydrogenase	EntrezGene identifier	Identity††%	Conserved††%
**L_1_ subcomplex (membrane-peripheral)**						
NuoB	AZC_1668	Nqo6	HyqI	AZC_4355	31	66
NuoC	AZC_1669	Nqo5	HyqG(N-term.)†	AZC_4356	23	57
NuoD	AZC_1670	Nqo4	HyqG(C-term.)‡		26	62
NuoE	AZC_1671	Nqo2				
NuoF	AZC_1672	Nqo1				
NuoG	AZC_1674	Nqo3				
NuoI	AZC_1676	Nqo9				
**L_O_ subcomplex (membrane-integral)**						
NuoA	AZC_1667	Nqo7				
NuoH	AZC_1675	Nqo8	HyqC	AZC_4359	23	58
NuoJ	AZC_1677	Nqo10	HyqE	AZC_4358	18	54
NuoK	AZC_1678	Nqo11				
NuoL	AZC_1679	Nqo12	HyqB	AZC_4360	24	55
NuoM	AZC_1680	Nqo13	HyqF	AZC_4357	22	61
NuoN	AZC_1667	Nqo14				

† 5′-end of *hyqG* encodes residues 1–156;

‡ 3′-end of *hyqG* encodes residues 157–504;

†† CLUSTAL 2.1 pairwise alignments.

### In Growth-optimized *A. caulinodans* Diazotrophic Liquid Cultures held at 20 µ*M* DOT, *endo-*hydrogenase Activity Serves *in vivo* as Respiratory Membrane e^–^ donor for Uptake of Endogenous H_2_

To recapitulate, *A. caulinodans* operates distinct, respiratory-membrane *exo*- and *endo*-hydrogenases; unlinked Δ*hyqRI*7 (*endo*-hydrogenase) and Δ*hupSL*2 (*exo-*hydrogenase) complete deletion alleles of relevant structural genes were previously isolated. In growth-optimized liquid diazotrophic cultures open to the environment, *exo*-hydrogenase mutants grow normally, whereas *endo*-hydrogenase mutants grow slowly [7]. To more accurately measure relative contributions of both *exo*- and *endo*-hydrogenase activities to *in vivo* recycling of H_2_ produced by Mo-dinitrogenase activity, H_2_ evolution rates of diazotrophic liquid batch cultures under continuous sparge have now been measured. *A. caulinodans* strains were batch cultured at 29°C in defined liquid media lacking utilizable-N; N_2_ as sole N-source was provided by continuous sparge with (2% O_2_, 5% CO_2_, bal. N_2_) gas mixture optimized for *A. caulinodans* N_2-_dependent growth (Materials). Dissolved O_2_ tension (DOT) in these sparged cultures held steady in the range of 18–20 µ*M* O_2_ as measured potentiometrically with a Clark-type polarographic electrode (Thermo-Orion 97–08). Culture exit gas streams were periodically sampled and analyzed for evolved H_2_ by gas chromatography (Materials). In these diazotrophic cultures, both *A. caulinodans* Δ*hyqRI* (*endo*-hydrogenase) mutant 66132 and Δ*hyqRI,* Δ*hupSL* (*exo*-, *endo*-hydrogenase) double-mutant 66204 showed tenfold elevated H_2_ evolution rates relative to both *hyq* ^+^, *hup* ^+^ parent 61305R and Δ*hupSL exo*-hydrogenase mutant 66081 (Table 2B).

**Table 2:** H_2_ evolution by *A. caulinodans* diazotrophic cultures.

*A. caulinodans*	Genotype	H_2_ evolved†	relative H_2_ evolved‡
**(A) N_2_ and NO_3_^–^ as N-sources (20 µM DOT)**			
66204	*ΔhyqRI ΔhupSL*	460	46.±5.0
66216R	*nifK ΔhupSL*	10	1.0±0.2
**(B) N_2_ as sole N-source (20 µM DOT; growth optimized)**			
61305R	*nif^+^ hyq^+^ hup^+^*	12	1.0±0.2
66081	*ΔhupSL*	16	1.3±0.3
66132	*ΔhyqRI*	175	15.±1.6
66204	*ΔhyqRI ΔhupSL*	540	45.±5.0
**(C) N_2_ as sole N-source (<1 µM DOT; microaerobic)**			
61305R	*nif^+^ hyq^+^ hup^+^*	7,100	1.0±0.2
66081	*ΔhupSL*	61,000	9.0±1.0
66132	*ΔhyqRI*	2,600	0.4±0.04
66204	*ΔhyqRI ΔhupSL*	14,000	2.2±0.4
**(D) N_2_ and NO_3_^–^ as N-source (<1 µM DOT; microaerobic)**			
60107R	*nifA*	1,100	1.0±0.2
66216R	*nifK ΔhupSL*	56,600	51.±3.0

† pmol 10^9^·cells^−1^ min^−1^ (typical, single experiment);

‡ multiple experiments.

Sparge rates for all liquid cultures were standardized to allow culture atmosphere exhaust rates of 0.5 min^–1^. In principle, for these growth-optimized diazotrophic cultures, relative abilities of *exo*- and *endo-*hydrogenases to recycle endogenous H_2_ might vary with sparge rates. Increased sparge rates proportionally decreased exit gas H_2_ levels of all cultures; relative H_2_ evolution rates among cultures were not affected. Culture sparges were slowed to the minimum rate still maintaining stable 20 µ*M* DOT. Nonetheless, Δ*hyqRI* single mutant 66132 still evolved tenfold more H_2_ than did Δ*hupSL exo*-hydrogenase mutant 66081. Because Δ*hyqRI* mutants invariably evolved more H_2_ than did Δ*hupSL* mutants at 20 µ*M* culture DOT, *endo-*hydrogenase activity is disproportionately responsible for recycling endogenous H_2_ produced by Mo-dinitrogenase activity in growth-optimized liquid cultures.

Similarly, when defined media were supplemented with 5 m*M* L-glutamine, measurable H_2_ evolution by all strains was negligible. In *A. caulinodans* cultures, L-glutamine sufficiency yields complete repression of the N_2_ fixation regulon, including *nifD^+^K^+^* genes encoding Mo-dinitrogenase [19] as well as *hyq* ^+^ genes encoding *endo*-hydrogenase [7]. *A. caulinodans* ORS571 wild-type also grows aerobically with either nitrate or nitrite as sole utilizable N-source; both nitrate (AZC0679) and nitrite (AZC0680–AZC0682) reductases are soluble and assimilatory; neither nitrate nor nitric oxide serves as respiratory e^–^ acceptor nor are these activities suggested by analysis of the complete genome sequence [20]. Similarly, *A. caulinodans* test strains were aerobically cultured in defined medium supplemented with 5 m*M* nitrate as utilizable N-source. Upon reaching a cell density of ∼1×10^8^ ml^–1^, exponentially growing liquid cultures were shifted to 2% O_2_ sparge and H_2_ levels of exit gases were monitored as before. In this protocol, H_2_ was evolved by *nifK* Δ*hupSL* double- mutant 66216R at baseline levels (Table 2A). By comparison, *exo*-, *endo*-hydrogenase double-mutant 66204 evolved H_2_ at levels corresponding to those of optimized diazotrophic cultures (Table 2A,2B). Accordingly, physiological H_2_ evolution at 20 µ*M* DOT was thus entirely owed to and benchmarked optimal Mo-dinitrogenase activity.

### In Microaerobic (<1 µ*M* DOT) *A. caulinodans* Cultures, *in vivo endo*-hydrogenase Activity Reverses, Driving Hydrogenic Respiratory Membrane e^–^ transfer at Extraordinarily High Rates

For *A. caulinodans* chemostat cultures sparged with 0.2% or more O_2_, elevated H_2_ production is not observed [21]. This critical O_2_ level corresponds to ≥0.9 µ*M* DOT, allowing *A. caulinodans* 57100 to be continuously cultured with succinate as C-source and N_2_ as N-source with O_2_ rate-limiting for growth [22]. In similar continuous cultures at <1 µ*M* DOT, *A. caulinodans* 57100 dinitrogenase activity levels decrease twofold [23] when compared to optimum (10–20 µ*M* DOT) diazotrophic culture conditions [21], [22], [23]. Accordingly, similar diazotrophic liquid batch cultures were initially sparged with 2% O_2_, 5% CO_2_, bal. N_2_ for 24 hr allowing cell densities to reach ∼1×10^8^ ml^–1^, at which point sparge gas O_2_ levels were decreased to 0.11%. In response, culture DOT levels declined precipitously, breaching 1 µ*M* DOT, true microaerobic physiology, defined as DOT insufficient to sustain conventional cytochrome *aa_3_* oxidase activity [2]. *A. caulinodans* microaerobic cultures employ two ultra-high O_2_ affinity terminal oxidases, cyt*cbb*_3_ and cyt*bd*, to maintain active oxidative phosphorylation [25]. When microaerobic cultures were supplemented with 5 m*M* L-glutamine and sampled periodically for viable cell counts by plating (Materials), all strains maintained exponential growth for 72+ hr; for all strains, microaerobic cell doubling-times were 8.1±1.5 hr at 29°C. Indeed, when strains were inoculated at low cell densities (∼1×10^6^ ml^–1^) and cultured microaerobically (0.11% O_2_ sparge) in minimal defined medium supplemented with 2.5 m*M* L-glutamine, all strains and cultures grew completely, and measurable H_2_ evolution in sparged culture exit gases was insignificant. Whereas, when microaerobic cultures were supplied with sparged (95%) N_2_ gas as sole N-source, no diazotrophy (cell doubling-times >20 hr) for any strain was measured. Neither was microerobic growth observed when cultures were supplemented with 5 m*M* nitrate or nitrite. When 5 m*M* ammonium was supplied, microaerobic growth of test strains was variable. Strain 60107R *nifA* and 66132 Δ*hyq* cultures both yielded cell-doubling times of 9.5±0.5 hr; parental strain 61305R cultures yielded cell-doubling times of 14±0.8 hr; for all other strains tested, cell-doubling times exceeded 20 hr. In all cases, microaerobic growth with ammonium as N-source inversely correlated with H_2_ evolution rates (Discussion).

Methylene blue (3,7-*bis*[dimethylamino]-phenothiazin-5-ium) serves as alternative e^–^ acceptor for respiratory complex I and, when reduced, as e^–^ donor to cyt*c*-dependent cytochrome oxidases, bypassing cytochrome *bc_1_* (respiratory complex III) activity and uncoupling oxidative phosphorylation [26]. Accordingly, methylene blue was deployed in microaerobic culture samples as *in vivo* respiratory e^–^ transfer probe. At experimentally sampled time points, culture samples were withdrawn into a gas tight syringe containing anoxic (colorless) methylene blue solution (2 µ*M* final); all culture samples initially turned visibly blue. However, when enclosed syringes were then held at 29°C, within 60 min all culture samples turned completely colorless (anoxic). When thus sampled, all microaerobic *A. caulinodans* cultures supplied excess succinate as C- and energy source retained respiratory-membrane e^–^ transfer activity for the duration of experiments (days).

Exit gas streams of sparged microaerobic cultures were sampled and H_2_ evolution was again measured by gas chromatography. Relative to optimized diazotrophic cultures (20 µ*M* DOT), H_2_ evolution of microaerobic (<1 µ*M* DOT), diazotrophic cultures dramatically increased. In parental 61305R cultures, microaerobic H_2_ evolution rates increased more than fiftyfold. For *ΔhupSL* (*exo*-hydrogenase) mutant 66081, H_2_ evolution rates increased almost four-thousandfold, which output persisted for 72+ hr. Yet, in *endo*-hydrogenase mutant 66132 cultures, H_2_ evolution rates increased only fifteen-fold (Table 2C). H_2_ evolution rates of all microaerobic batch cultures were sustained 72+ hr given sufficient oxidizable organic-C (succinate) as energy substrate. In conclusion, the extraordinarily high H_2_ evolution rates of strain-specific microaerobic cultures required both *endo*-hydrogenase present and *exo*-hydrogenase absent.

Recall that Mo-dinitrogenase operates in concert with *endo*-hydrogenase activity given optimum (20 µ*M* DOT) diazotrophic physiology. To what extent does Mo-dinitrogenase activity contribute to H_2_ output by microaerobic (<1 µ*M* DOT) cultures? To test this hypothesis, similar microaerobic shift experiments were conducted with cultures grown with and maintained on 5 m*M* nitrate as N-source, which allows full transcriptional derepression of the N_2_ fixation regulon [19]. Upon microaerobic shift, nitrate-grown *nifK* Δ*hupSL* double-mutant 66216R likewise showed exceedingly high H_2_ output (Table 2D). Note that for all strains, microaerobic cultures with nitrate fail, implying nitrate is not then a competing e^–^ acceptor. Moreover, nitrate itself has no inducible effect on H_2_ evolution; *nifA* null mutant 60107R, which entirely lacks ability to derepress the N_2_ fixation regulon [19], shows negligible H_2_ output in microaerobic nitrate-supplemented culture (Table 2D). Thus, Mo-dinitrogenase activity itself contributes little (<10%) of the exceedingly high H_2_ output by Δ*hupSL* mutant microaerobic cultures. Moreover, because Δ*hyqRI* Δ*hupSL* double-mutant 66204 retains 20% microaerobic H_2_ evolution rates when compared to Δ*hupSL* single-mutant 66081 (Table 2C), an additional, uncharacterized H_2_ source is then operative. As it is absent in *nifA* mutant 60107R (Table 2D), this additional microaerobic H_2_ source also seems associated with the N_2_ fixation regulation. In summary, *endo-*hydrogenase activity is itself responsible for ∼80% of the H_2_ evolved, *i.e.*, net hydrogenic respiratory membrane e^–^ transfer rates, by microaerobic cultures in which the N_2_ fixation regulon is derepressed.

## Discussion

In summary, *A. caulinodans endo*-hydrogenase is a bidirectional catalyst whose *in vivo* activity reverses in response to physiological O_2_ availability. In optimized diazotrophic (20 µ*M* DOT) cultures, *endo*-hydrogenase operates in H_2_ uptake mode, consuming endogenous H_2_ as respiratory e^–^ donor. In microaerobic (<1 µ*M* DOT) cultures, membrane-integral *endo*-hydrogenase switches to hydrogenic respiratory-membrane e^–^ transfer mode, employing H^+^ ions as terminal e^–^ acceptor. Accordingly, *endo*-hydrogenase shares a microaerobic terminal oxidase role with cytochrome *cbb*_3_ and cytochrome *bd* [25], whereas respiratory complexes I (NADH:quinone oxidoreductase) and II (succinate dehydrogenase) serve as net e^–^ donors. Measured *in vivo* respiratory-membrane *endo*-hydrogenase H_2_ production rates (45,000 pmol 10^9^·cells^−1^ min^−1^) are orders of magnitude higher than previously observed for O_2_ tolerant hydrogenases in non-fermentative microorganisms. In *A. caulinodans exo*-hydrogenase mutants lacking microaerobic H_2_ uptake activity, respiratory membrane *endo*-hydrogenase mediated H_2_ production in liquid batch cultures persists at high rates for 72+ hr. Sum totals of evolved H_2_ (2e^–^ reduction) at 72 hr are 230±30 µmol per 10^9^ cells, representing net oxidation of some 25% of total (340 µmol) succinate supplied these cultures as sole organotrophic energy source and quantitatively converted to *poly*-β-hydroxbutyrate as organic end-product (5e^–^ oxidation per succinate; [24], [27]).

Similarly, hydrogenic Mo-dinitrogenase activity, at most 10% of hydrogenic *endo*-hydrogenase activity, consumes almost tenfold more NADH on a mole:mole basis (4 NADH for reductant; 5+ NADH as substrate for oxidative phosphorylation to make the required 16 ATP) [10], [11]. Then, Mo-dinitrogenase activity itself consumes similar amounts of succinate. Unsurprisingly, for all strains tested, diazotrophic (N_2_ as sole N-source) microaerobic liquid batch cultures open to the environment fail (cell doubling-times >20 hr). Whereas, all strains may be successfully cultured microaerobically with L-glutamine provided as N-source absent all hydrogenesis. When provided ammonium and N_2_ as N-sources, some strains grow microaerobically, albeit slowly as significant N_2_ fixation persists; any growth inversely correlates with hydrogenesis by diazotrophic microaerobic cultures (Table 2C). When atmospheric N_2_ is entirely replaced by argon, ammonium-supplemented microaerobic cultures indeed grow [27], hence Mo-dinitrogenase activity is explicitly responsible for failed microaerobic growth. Earlier, we reported *A. caulinodans* microaerobic diazotrophic liquid suspension cultures showed increased spectrophotometric absorbance at 600 nm [25]. In more recent experiments, growth in microaerobic diazotrophic liquid suspension cultures was measured by removing samples and aerobically plating for viable cell counts on rich media (Materials) as, in these cultures, viable cell counts do not correlate with increased spectrophotometric absorbance at 600 nm. Likewise, colony growth tests on solid media reflect multiple cell physiology states, and colony growth is facilitated by more efficient H_2_ recycling at increased cell densities [12].

Among bacteria with known genome sequences, eight genera (A. caulinodans, Azospirillum brasilense, Beijerinckia indica, Bradyrhizobium japonicum; Rhizobium leguminosarum bv. viciae, Rhodopseudomonas palustris, Rhodocista centenaria, Xanthobacter autotrophicus), all microaerophiles capable of N_2_ fixation, possess orthologous hyq^+^ operons encoding endo-hydrogenase [12]. Of the six, inferred endo-hydrogenase subunits, five have close homologs in L-type respiratory complex I (Table 1). The sixth (HyqG) protein is homologous to a fused NuoC/D protein. The presumed HyqG H_2_ catalytic site shared among conserved group-4 hydrogenases is yet undetermined. The group-4 HyqG superfamily (SSF56762) also includes the group-1 exo-hydrogenase catalytic subunit, which possesses a heteronuclear Ni,Fe catalytic center coordinated by four, completely conserved Cys residues, of which two bridge the catalytic Ni and Fe = C = O binuclear center [28]. To the contrary, inferred HyqG proteins from eight microaerophilic genera all lack both N-terminal and C-terminal Cys-X-X-Cys motifs. Rather, three Cys residues (A. caulinodans Cys-258, Cys-491, and Cys-497) are completely conserved by the HyqG family (Fig. S7). The NuoD (Nqo4) proteins of bacterial respiratory complex I, also members of this same Superfamily, neither possess the Cys-X-X-Cys pairs nor do they exhibit a Ni,Fe binuclear center. Therefore, that HyqG actually carries a binuclear Ni,Fe catalytic site seems uncertain, if not unlikely. The binding site for respiratory complex I membrane ubiquinone, its ultimate e^–^ acceptor, is a cavity formed between a four-helix bundle of NuoD (Nqo4), the H1 helix of NuoB (Nqo6), and transmembrane helix 1 of NuoH (Nqo8) [14], [18], all of which motifs are conserved in the Hyq endo-hydrogenase (HyqG, HyqI, and HyqI, respectively).

By inference, the Hyq *endo*-hydrogenase is a membrane-integral H_2_:quinone oxidoreductase. Given the strong reducing potential of the biochemical standard hydrogen electrode (*E*_o_′ = –0.414*V*) relative to that of ubiquinone (*E*_o_′ = +0.070*V)* respiratory-membrane hydrogenesis is a highly endergonic process under standard conditions. In *Rps. palustris*, membrane physiology has been modeled under a variety of dynamic steady-state conditions including microaerobic respiration, whose membrane ubiquinone pools are necessarily highly (>90%) reduced [29]. If membrane ubiquinone pools were poised some 90% reduced, a ubiquinon/ubiquinol half-cell potential (*E*′) of +0.040*V* (at 25°C) would obtain. By inference, the balanced reaction for *endo*-hydrogenase mediated H_2_ uptake, including trans-membrane H^+^ pumping, may be written:
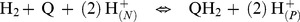
(1)where *N* connotes *endo* and *P* connotes *exo* membrane faces. In purified, reconstituted vesicles, respiratory complex I activity, including H^+^ translocation, is fully reversible [30]. If the same holds true for *endo*-hydrogenase *in vivo*, its hydrogenesis mode activity may be written:



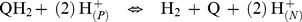
(2)This activity would tap steady-state membrane proton-motive force (*Δp*), modeled in *Rps. palustris* microaerobic respiratory membranes as *Δp* = 0.195*V* [29]. Were 2H^+^(*_P_*) counter-transported during steady-state hydrogenesis, *E*′ values at 25°C would be effectively lowered from +0.040*V* to −0.350*V*, at which steady-state potential the operative hydrogen half-cell H_2_ partial pressure (*p*H_2_) would approach 0.7 kPa at 25°C. Indeed, when exit gases of sparged *A. caulinodans* hydrogenic cultures maintained at 30°C were analyzed, *p*H_2_ levels reproducibly approached 0.7 kPa as sustained hydrogenesis rates. In all likelihood, high-level *endo*-hydrogenase dependent H_2_ production requires both highly reduced membrane ubiquinone pools and high *Δp* values. In *A. caulinodans* microaerobic liquid batch cultures, elevated H_2_ production requires supplementation with excess, primary C-source (succinate, L-malate, or L-lactate), which presumably drive reduction of respiratory membrane ubiquinone pools at relatively high rates. Moreover, if indeed consumptive of membrane *Δp*, respiratory membrane hydrogenesis only operates when sufficient O_2_ is also available as respiratory e^−^ acceptor to regenerate high *Δp* (respiratory complex I, III, and IV activities). Regardless, any respiratory-membrane hydrogenesis would necessarily largely uncouple oxidative phosphorylation.

For *A. caulinodans* microaerobic respiration, both available (limiting) O_2_ and H^+^ ions simultaneously serve as e^–^ acceptors. *A. caulinodans* then employs multiple cyt*c*- and ubiquinol-oxidases [25], resulting in varied respiratory membrane proton-translocation yields. So, no fixed stoichiometries of H_2_ relative to H_2_O production may be deduced. Moreover, parceling out relative *in vivo* contributions as respiratory membrane e^–^ acceptors is problematic, given restricted choice. When one or more e^–^ acceptor activities are absent due to mutation, compensatory flux to soluble e^–^ acceptors, such as N_2_ (Mo-dinitrogenase), NO_3_^−^ (nitrate reductase), and CO_2_ (both rubisco and CO dehydrogenase) then obtains. As one example, H_2_ evolution rates for *exo*- *endo*-hydrogenase double-mutant 66204 increased twenty-five-fold when shifted from growth optimal (20 µ*M* DOT) to microaerobic (<1 µ*M* DOT) conditions, presumably owed to restricted choice of available e^–^ acceptors (*i.e.*, relative absence of O_2_). Moreover, restricted choice also extends to e^–^ donors. Because it successfully reoxidizes >80% of H_2_ then produced by combined (>90%) *endo*-hydrogenase and (<10%) Mo-dinitrogenase activities, *exo*-hydrogenase operates as a relatively more competitive microaerobic respiratory membrane e^–^ donor. Conceivably, these changes in cellular microaerobic respiratory membrane physiology might simply reflect more-reduced ubiquinone pools. Alternatively, respiratory membranes might build *exo*- and/or *endo*-hydrogenases into macromolecular complexes which would preclude simple diffusion control of respiratory e^−^ transfer by membrane ubiquinone pools.

In *A. caulinodans*, the two, respiratory membrane hydrogenases possess distinct physiological roles; group-4 *endo*-hydrogenase activity is bidirectional and strictly correlates with diazotrophy and endogenous H_2_ uptake, whereas group-1 *exo-*hydrogenase activity is unidirectional and also allows chemoautotrophy with exogenous H_2_ as energy source [7]. Among capable anaerobes, fermentative membrane hydrogenesis is well described [1], [2], [5]. Whereas, among obligate aerobes, respiratory membrane hydrogenesis as a sustained physiological process seems counterproductive, as it significantly uncouples oxidative phosphorylation. Indeed, in diazotrophic microaerobic *A. caulinodans* cultures, *exo*-hydrogenase and *endo*-hydrogenase are both highly active and thus seemingly operative at cross-purposes.

However, liquid culture experiments, which strive to allow all bacterial cells a similar physiological milieu, are contrived. In reality, bacterial cell populations experience a dimensional world. We suggest, as one possibility, concomitant H_2_ evolution and H_2_ uptake might prove useful if partitioned among aerobic and microaerobic bacterial cells in dimensional populations. Varying O_2_ microenvironments within organotrophic bacterial colonies or biofilms might *de facto* segregate metabolic physiology, allowing internal O_2_-restricted cells to evolve H_2_ and external O_2_-sufficient cells to take up and use that H_2_, driving oxidative phosphorylation. Superficial H_2_ oxidizing, O_2_ rich cells might then redirect environmental organic-C sources away from catabolism (oxidative phosphorylation substrate) towards anabolism (C-assimilation), augmenting growth rates and proliferation of dimensionally organized and specialized cell populations considered as a whole.

## Materials and Methods

### Bacterial Strains and Media

*Azorhizobium caulinodans* ORS571 wild-type (strain 57100; ATCC No. 43989), was originally isolated from *Sesbania rostrata* stem-nodules [8]. Strain 61305R [32], a 57100 derivative carrying an IS*50*R insertion in the (catabolic) nicotinate dehydrogenase structural gene served as ‘virtual’ wild-type for reported experiments; 61305R uses supplied (3 µ*M*) nicotinate only as anabolic substrate for synthesis of pyridine nucleotides, for which 57100 is auxotrophic [33]. Precise, in-frame deletion mutagenesis of *A. caulinodans* target genes was conducted out by “crossover PCR” as previously described [34]. Strain 66216R was constructed as for strain 66081 with strain 60057R as parent (Table 3). Defined media for all cultures was basal NIF medium (7.5 m*M* potassium phosphate pH 6.3, 1 m*M* MgSO_4_, 0.5 m*M* CaCL_2_, 2 µ*M* ferric citrate, 3 µ*M* nicotinate, 1 µ*M* sodium molybdate, 1 µ*M* pantothenate, 0.1 µ*M* D-biotin, and Hutner’s “44′ trace elements [35]) supplemented with 20 m*M* potassium succinate as sole C- and energy source, and 2.5 m*M* ammonium bicarbonate as N-source. Strains (whose lineage does not include 61305R) and which actively catabolize nicotinate were supplemented with 0.1 m*M* nicotinate in aerobic cultures; in microaerobic cultures, *A. caulinodans* wild-type 57100 does not measurably catabolize nicotinate.

**Table 3:** *Azorhizobium caulinodans* strains.

Strain	Genotype	Ref.
57100	ORS571 wild-type	[8]
60035	57100 *nifD35::Vi*	[31]
60035R	60035 *nifD35*::IS*50*R	
60057R	60057 *nifK57*::IS*50*R	
60107R	57100 *nifA107R*	
61305R	57100 Nic^–^, 6-OH-Nic^+^	[32]
66081	61305R *hup*Δ*SL*2	[7]
66132	61305R *hyqΔRI*7	[7]
66204	61305R *hup*Δ*SL*2 *hyqΔRI*7	[7]
66216R	60057R *hup*Δ*SL*2	

### Physiological Growth Measurements and Evolved H_2_ Analyses

Starter cultures of *A. caulinodans* strain 61305R and its derivatives were aerobically cultured in minimal defined NIF liquid medium [8] supplemented with: 0.3 m*M* ammonium as sole, limiting N-source and 3 µ*M* nicotinate at 37°C until growth arrest (cell densities ∼1×10^8^ cells ml^−1^). For kinetic measurements of diazotrophy, arrested starter cultures were each diluted one-hundredfold in 20 ml NIF medium; serum vials (30 ml capacity) were sealed with silicone rubber septa, sparged continuously (10 ml min^−1^) with defined gas mixtures (*e.g.* 2% O_2_, 5% CO_2_, bal. N_2_), and incubated at 29°C. At least three times per cell-doubling period, culture samples were removed, serially diluted, plated on rich GYPC medium [9], and incubated aerobically 48 hr at 37°C; colonies were counted in triplicate. *In vivo* H_2_ uptake activities were inferred, coupled to Mo-dinitrogenase activity as H_2_ donor, by comparing rates of H_2_ evolution from *A. caulinodans hup* ^+^ *hyq* ^+^ (wild-type), Δ*hupSL* (*exo*-hydrogenase) mutant, Δ*hyqRI* (*endo*-hydrogenase) mutant, and Δ*hupSL* Δ*hyqRI* double-mutant cultures (Table 3). Collectively, both hydrogenases account for all *in vivo* H_2_ uptake activity [12]. (Amperometric cell-free assay of isolated *endo*-hydrogenase H_2_ uptake activity with exogenous H_2_ as substrate is not at hand.) To measure evolved H_2_, sparged culture exit gas streams were sampled and analyzed by gas chromatography (RPC1; Peak Laboratories LLC) fitted with an HgO (reducing compound) photometer as detector [36] and a fixed volume (25 µl) sampling loop. Molar H_2_ evolution rates were inferred from measured dilution rates of culture atmospheric volumes. Total cellular protein was measured by the bicinchoninic acid procedure (Sigma-Aldrich Co.); for *A. caulinodans* and related microaerophiles employing oxidative metabolic gearing, mean total cell proteins levels (135±15 fg) are largely independent of cell physiology [24].

## Supporting Information

Figure S1 ***A. caulinodans*** **HyqB and NuoL (CLUSTAL W2) alignment.**
(EPS)

Figure S2 ***A. caulinodans*** **HyqC and NuoH (CLUSTAL W2) alignment.**
(EPS)

Figure S3 ***A. caulinodans*** **HyqE and NuoJ (CLUSTAL W2) alignment.**
(EPS)

Figure S4 ***A. caulinodans*** **HyqF and NuoM (CLUSTAL W2) alignment.**
(EPS)

Figure S5 ***A. caulinodans*** **HyqG and NuoC/D (CLUSTAL W2) alignment.**
(EPS)

Figure S6 ***A. caulinodans*** **HyqI and NuoB (CLUSTAL W2) alignment.**
(EPS)

Figure S7 **HyqG orthologs (CLUSTAL W2) multiple alignment.** From the top, species are: *Rhizobium leguminosarum* (Rhleg), *Azorhizobium caulinodans* (Azoca), *Xanthobacter autotrophicus* (Xanpy2), *Beijerinckia indica* (Beind), *Bradyrhizobium japonicum* (Braja), *Rhodospirillum centenum*, (Rhoce), *Rhodopseudomonas palustris* (BisA53), *Azospirillum brasilense* (Azobr), *Rhodopseudomonas palustris* (HaA2), *Rhodopseudomonas palustris* (BisB18), *Rhodopseudomonas palustris* (BisB5).
(EPS)
